# Supplementary material for: Comparative in-vivo bond failure rate of orthodontic brackets when bracket base is treated with micro-abrasive blasting vs. acid etching: eighteen month randomized control trial and scanning electron microscope study
Source: PeerJ. 2024 Jun 28;12:e17645. doi: 10.7717/peerj.17645 (PMC11216187; doi:10.7717/peerj.17645)
Supplement: Supplemental Information 3 [file peerj-12-17645-s003.doc]

**Completed STROBE checklist**

This checklist was elaborated using formal items recommended for cross-sectional studies from STROBE statement (https://www.strobe-statement.org).

|  | Item No | Recommendation | Respected ? | Comments and quotes | Section /Page no. |
| --- | --- | --- | --- | --- | --- |
| **Title and abstract** | 1 | (*a*) Indicate the study’s design with a commonly used term in the title or the abstract | Yes | Study design is indicated in the Methods section of the abstract.  “This is a randomized control trial” | Abstract, Page-3, line no. 49 |
|  |  | (*b*) Provide in the abstract an informative and balanced summary of what was done and what was found | Yes | This information are stated in the study abstract (study objective described, method and results described) | Abstract, Page-3, line no. 50 |
| Introduction | | |  |  |  |
| Background/rationale | 2 | Explain the scientific background and rationale for the investigation being reported | Yes | Rationale and existing literature are stated in the introduction section | Introduction,  Page-4  Line no 74-90 |
| Objectives | 3 | State specific objectives, including any prespecified hypotheses | Yes | A statement at the end of the introduction specifies the specific goals and objectives. The aims of this randomized control trial were: (1) To describe a method to chemically etch the bases of stainless-steel orthodontic brackets. (2) To observe the changes in an electron microscope in the microstructure of the brackets with sandblasted bases and acid-etched bases, and (3) To compare the survivability of orthodontic brackets with a chemical etched base versus a sandblasted base bonded with Transbond XT using the conventional acid etch technique, over a follow-up of 18 months. | Introduction,  Page-4  Line no.91 - 96 |
| Methods | | |  |  |  |
| Study design | 4 | Present key elements of study design early in the paper | Yes | Study design is stated in the first subsection of Methods. Key elements are all described in the methods.  This split-mouth study was a randomized clinical control trial with triple-blinding. | Methods,  Page-5  Line no. 98 |
| Setting | 5 | Describe the setting, locations, and relevant dates, including periods of recruitment, exposure, follow-up, and data collection | Mostly | Setting, contexts, dates of inclusion, are fully described in the method section .  Written consent of the patients and the approval of the Ethical Committee of Islamic International Dental Hospital, Riphah International University where the study was conducted were taken prior to the commencement of this study | Methods,  Page-5  Line no.101 -103 |
| Participants | 6 | (*a*) Give the eligibility criteria, and the sources and methods of selection of participants | Yes | Study population is described is the method section, as well as selection criteria  “patients with permanent dentition planned to undergo a minimum of 18 months of orthodontic therapy and bond up of the brackets with the conventional acid etch technique.” | Methods,  Page-5  Line no.105 – 107 |
| Variables | 7 | Clearly define all outcomes, exposures, predictors, potential confounders, and effect modifiers. Give diagnostic criteria, if applicable | Yes | Standardized variable definitions were used across all programs, which are presented in method section. | Methods,  Page-7  Line no.162 |
| Data sources/ measurement | 8* | For each variable of interest, give sources of data and details of methods of assessment (measurement). Describe comparability of assessment methods if there is more than one group | Yes | Data collection and measurement was the same for all variables, and is described in the methods section.  “The main outcome measure was the debonding/failure of the bracket”. | Methods,  Page-7  Line no.162 |
| Bias | 9 | Describe any efforts to address potential sources of bias | Yes | We notably tried to reduce bias by a triple blind study design.  The analysis section also explains this part. | Methods,  Page-7  Line no.163 - 168 |
| Study size | 10 | Explain how the study size was arrived at | Yes | The sample size was estimation before the study accordingly. | Methods,  Page- 7  Line 143 - 154 |
| Quantitative variables | 11 | Explain how quantitative variables were handled in the analyses. If applicable, describe which groupings were chosen and why | Yes | Definitions of all categories for variables are presented method section. | Methods,  Page-7  Line no.162 |
| Statistical methods | 12 | (*a*) Describe all statistical methods, including those used to control for confounding | Yes | The data was analyzed with the computer software SPSS (version 20, IBM). Kaplan-Meier survival analysis was performed, and cumulative survival curves and log survival curves were plotted along with a statistical comparison of the two groups with the log-rank (Mantel-Cox) test. | Methods,  Page-8  Line no.170 - 173 |
|  |  | (*b*) Describe any methods used to examine subgroups and interactions | N/A | Not Applicable |  |
|  |  | (*c*) Explain how missing data were addressed | N/A | Not Applicable |  |
|  |  | (*d*) If applicable, describe analytical methods taking account of sampling strategy | N/A | Not applicable | - |
|  |  | (*e*) Describe any sensitivity analyses | N/A | Not applicable | - |
| Results | | |  |  |  |
| Participants | 13* | (a) Report numbers of individuals at each stage of study—eg numbers potentially eligible, examined for eligibility, confirmed eligible, included in the study, completing follow-up, and analysed | Yes | A total of 310 patients participated in the study | Results,  Page-8  Line no.177 |
|  |  | (b) Give reasons for non-participation at each stage | N/A | Not Applicable |  |
|  |  | (c) Consider use of a flow diagram | Yes | COSORT Flow chart is given | - |
| Descriptive data | 14* | (a) Give characteristics of study participants (eg demographic, clinical, social) and information on exposures and potential confounders | Yes | Care was taken to not to include patients with gross deep bites or crossbites affecting bracket positioning, any enamel abnormalities, cavitation or fillings/restorations including crowns on the buccal surface, patients previously treated with fixed orthodontic appliances, or patients who had dental bleaching prior to the commencement of their orthodontic treatment and patients in which rapid expander or a fixed functional appliance was planned. | Results,  Page-5  Line 113-118 |
|  |  | (b) Indicate number of participants with missing data for each variable of interest | N/A |  |  |
| Outcome data | 15* | Report numbers of outcome events or summary measures | Yes | All numbers are reported in the results section | Results,  Page-8  Line 177-186 |
| Main results | 16 | (*a*) Give unadjusted estimates and, if applicable, confounder-adjusted estimates and their precision (eg, 95% confidence interval). Make clear which confounders were adjusted for and why they were included | N/A | N/A | - |
|  |  | (*b*) Report category boundaries when continuous variables were categorized | N/A |  |  |
|  |  | (*c*) If relevant, consider translating estimates of relative risk into absolute risk for a meaningful time period | N/A | N/A | - |
| Other analyses | 17 | Report other analyses done—eg analyses of subgroups and interactions, and sensitivity analyses | N/A | N/A | - |
| Discussion | | |  |  |  |
| Key results | 18 | Summarise key results with reference to study objectives | Yes | Key results are described at the beginning of discussion section, and later on by the mean of a paragraph displaying main operational implications. They also are summarized in the conclusion | Discussion,  Page-10  Line, 218 - 220 |
| Limitations | 19 | Discuss limitations of the study, taking into account sources of potential bias or imprecision. Discuss both direction and magnitude of any potential bias | Yes | Description of limitations is done in the discussion section. | Discussion,  Page-11  Line, 248 - 250 |
| Interpretation | 20 | Give a cautious overall interpretation of results considering objectives, limitations, multiplicity of analyses, results from similar studies, and other relevant evidence | Yes | References were added where possible and discussed. Limitations were taken into account in the discussion. | Discussion,  Page-9  Line, 192-250 |
| Generalisability | 21 | Discuss the generalisability (external validity) of the study results | Yes | Study generalizable described in the discussion section. | Discussion,  Page-11  Line, 249-250 |
| Other information | | |  |  |  |
| Funding | 22 | Give the source of funding and the role of the funders for the present study and, if applicable, for the original study on which the present article is based | N/A | Funding information were displayed upon submission but not included in the manuscript, as requested. | - |
|  | | |  |  |  |

*Give information separately for exposed and unexposed groups.
