# Supplementary figures and images for: Comparative in-vivo bond failure rate of orthodontic brackets when bracket base is treated with micro-abrasive blasting vs. acid etching: eighteen month randomized control trial and scanning electron microscope study
Source: PeerJ. 2024 Jun 28;12:e17645. doi: 10.7717/peerj.17645 (PMC11216187; doi:10.7717/peerj.17645)

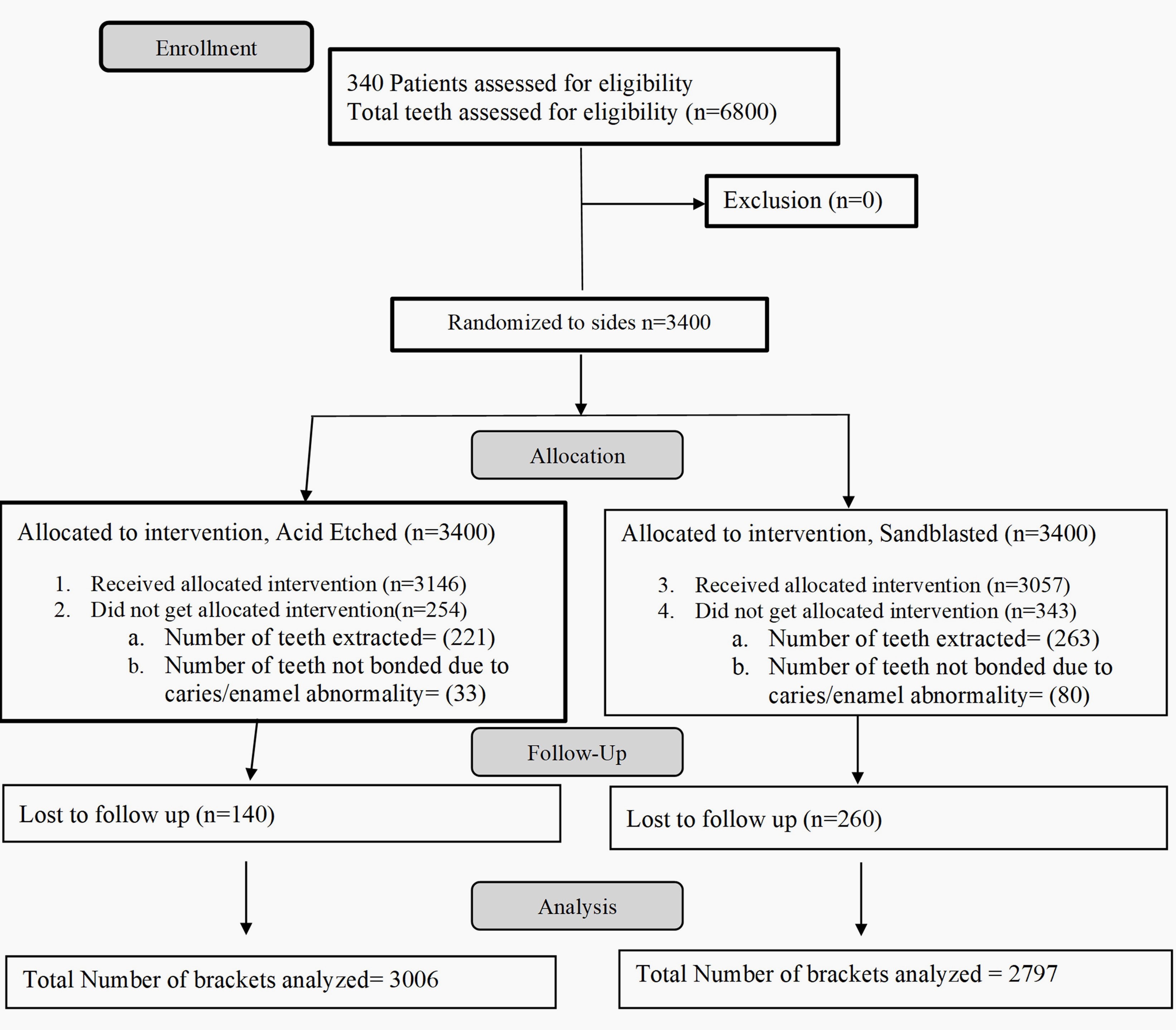

Supplement: Supplemental Information 4 [file peerj-12-17645-s004.jpeg]
