# Supplementary material for: Comparative in-vivo bond failure rate of orthodontic brackets when bracket base is treated with micro-abrasive blasting vs. acid etching: eighteen month randomized control trial and scanning electron microscope study
Source: PeerJ. 2024 Jun 28;12:e17645. doi: 10.7717/peerj.17645 (PMC11216187; doi:10.7717/peerj.17645)
Supplement: Supplemental Information 5 [file peerj-12-17645-s005.docx]

**Supplementary Materials**

**Questionnaire used in the study**

**Question SPSS data code**

A1. The tooth FDI Number 11, 12, 13, 14, 15,

21, 22, 23, 24, 25

31, 32, 33, 34, 35

41, 42, 43, 44, 45

A2. Time in Months

Numeric values from 1 to 18

A3. Failure of Bracket

a. Censor (0)

b. Failure (1)

A4. Group

a. Sandblast (1)

b. Acid Etch (2)
