# Supplementary material for: Comparative in-vivo bond failure rate of orthodontic brackets when bracket base is treated with micro-abrasive blasting vs. acid etching: eighteen month randomized control trial and scanning electron microscope study
Source: PeerJ. 2024 Jun 28;12:e17645. doi: 10.7717/peerj.17645 (PMC11216187; doi:10.7717/peerj.17645)
Supplement: Supplemental Information 6 [file peerj-12-17645-s006.docx]

**Trail Protocol of this Randomized Controlled Trial**

**Title:** Comparative in-vivo bond failure rate of orthodontic brackets when bracket base is treated with Micro-abrasive blasting vs Acid Etching: Eighteen Month Randomized Control Trial and Scanning Electron Microscope Study

**Protocol Version: 1
Date: 01/06/20**

**1. Study Team**

Principal Investigator: Owais Khalid Durrani
Co-Investigators: Ulfat Bashir Raja , Farooq Ahmad Chaudhary ,Umar Hamid , Muhammad

Qasim Javed, Sundus Atique , Syed Rashid Habib
Study Coordinator: Owais Khalid Durrani
Data Manager: Farooq Ahmad Chaudhary
Statistical Advisor: Farooq Ahmad Chaudhary

**2. Study Objectives**

Primary Objective: [To compare the survivability of orthodontic brackets with a chemical etched base versus a sandblasted base bonded with Transbond XT using the conventional acid etch technique, over a follow-up of 18 months.]
Secondary Objectives: [(1) To describe a method to chemically etch the bases of stainless-steel orthodontic brackets. (2) To observe the changes in an electron microscope in the microstructure of the brackets with sandblasted bases and acid-etched bases]

**3. Study Design**

3.1 Study Type: Randomized Controlled Trial
3.2 Randomization: Computer generated
3.3 Blinding: Triple Blind
3.4 Study Duration: 18 months
3.5 Sample Size: 310 patients

**4. Participants**

4.1 Inclusion Criteria: [patients with permanent dentition planned to undergo a minimum of 18 months of orthodontic therapy and bond up of the brackets with the conventional acid etch technique]
4.2 Exclusion Criteria: [patients with gross deep bites or crossbites affecting bracket positioning, any enamel abnormalities, cavitation or fillings/restorations including crowns on the buccal surface, patients previously treated with fixed orthodontic appliances, or patients who had dental bleaching prior to the commencement of their orthodontic treatment and patients in which rapid expander or a fixed functional appliance was planned]
4.3 Recruitment: Convenience sampling

**5. Interventions**

5.1 Experimental Group: Brackets with Acid etched base
5.2 Control Group: Brackets with sandblasted base
5.3 Dose and Duration: Eighteen months, dose and frequency not applicable

**6. Outcomes**

6.1 Primary Outcome: failure of brackets 18 months after debonding
6.2 Secondary Outcomes: none

**7. Data Collection**

7.1 Data Sources: clinical observation, electron microscope images
7.2 Data Collection Tools: Scanning electron microscope
7.3 Data Management: Data managed in the SPSS Software

**8. Statistical Analysis**

8.1 Statistical Methods: Log rank test and Kaplan Meir plot curve to compare the two groups
8.2 Interim Analysis: Specify if interim analyses will be conducted and under what circumstances

**9. Ethical Considerations**

9.1 Informed Consent: written consent was obtained from all the participants
9.2 Ethics Committee Approval: Ethical Committee of Islamic International Dental Hospital, Riphah International University (IIDC/IRC/06/06/2020).

**10. Funding**

10.1 Funding Source: None
10.2 Conflicts of Interest: None

**11. Dissemination Plan**

publications, conferences

**12. Amendments**

None

**13. References**

[Algera TJ, Kleverlaan CJ, Prahl-Andersen B, and Feilzer AJ. 2008. The influence of different bracket base surfaces on tensile and shear bond strength. Eur J Orthod 30:490-494. 10.1093/ejo/cjn029

Buonocore MG. 1955. A simple method of increasing the adhesion of acrylic filling materials to enamel surfaces. J Dent Res 34:849-853.

Chaudhary FA, Ahmad B, Butt DQ, Hameed S, and Bashir U. 2019. Normal range of maximum mouth opening in pakistani population: A cross-sectional study. Journal of International Oral Health 11:353.

Dickinson PT, and Powers JM. 1980. Evaluation of fourteen direct-bonding orthodontic bases. Am J Orthod 78:630-639.

Din SU, Sajid M, Saeed A, Chaudhary FA, Alam MK, Sarfraz J, Ahmed B, and Patel M. 2022. Dimensional changes of commercial and novel polyvinyl siloxane impression materials following sodium hypochlorite disinfection. PeerJ 10:e12812.

Kirkpatrick JJ, Enion DS, and Burd DA. 1995. Hydrofluoric acid burns: a review. Burns 21:483-493.

Linklater RA, and Gordon PH. 2003. Bond failure patterns in vivo. Am J Orthod Dentofacial Orthop 123:534-539. 10.1067/mod.2003.S0889540602000252

Lugato IC, Pignatta LM, Arantes Fde M, and Santos EC. 2009. Comparison of the shear bond strengths of conventional mesh bases and sandblasted orthodontic bracket bases. Braz Oral Res 23:407-414.

MacColl GA, Rossouw PE, Titley KC, and Yamin C. 1998. The relationship between bond strength and orthodontic bracket base surface area with conventional and microetched foil-mesh bases. Am J Orthod Dentofacial Orthop 113:276-281.

Maijer R, and Smith DC. 1981. Variables influencing the bond strength of metal orthodontic bracket bases. Am J Orthod 79:20-34.

McKee D, Thoma A, Bailey K, and Fish J. 2014. A review of hydrofluoric acid burn management. Plast Surg (Oakv) 22:95-98.

Millett D, McCabe JF, and Gordon PH. 1993. The role of sandblasting on the retention of metallic brackets applied with glass ionomer cement. Br J Orthod 20:117-122.

Murray SD, and Hobson RS. 2003. Comparison of in vivo and in vitro shear bond strength. Am J Orthod Dentofacial Orthop 123:2-9.

Newman G. 1965. Epoxy adhesives for orthodontic attachments: progress report. Am J Orthod 51:901-912.

Newman GV, Snyder WH, and Wilson CE, Jr. 1968. Acrylic adhesives for bonding attachments to tooth surfaces. Angle Orthod 38:12-18.

Oilo G. 1992. Biodegradation of dental composites/glass-ionomer cements. Adv Dent Res 6:50-54. 10.1177/08959374920060011701

Ozer M, and Arici S. 2005. Sandblasted metal brackets bonded with resin-modified glass ionomer cement in vivo. Angle Orthod 75:406-409.

Ozer M, Bayram M, Dincyurek C, and Tokalak F. 2014. Clinical bond failure rates of adhesive precoated self-ligating brackets using a self-etching primer. Angle Orthod 84:155-160. 10.2319/022013-149.1

Pandis N, Polychronopoulou A, and Eliades T. 2011. Sample size estimation: an overview with applications to orthodontic clinical trial designs. Am J Orthod Dentofacial Orthop 140:e141-146. 10.1016/j.ajodo.2011.04.021

Pocock SJ. 1983. Clinical Trials: A Practical Approach: Wiley.

Sharma-Sayal SK, Rossouw PE, Kulkarni GV, and Titley KC. 2003. The influence of orthodontic bracket base design on shear bond strength. Am J Orthod Dentofacial Orthop 124:74-82.

Shyagali TR, Bhayya DP, Urs CB, and Subramaniam S. 2015. Finite element study on modification of bracket base and its effects on bond strength. Dental Press J Orthod 20:76-82. 10.1590/2176-9451.20.2.076-082.oar

Smith NR, and Reynolds IR. 1991. A comparison of three bracket bases: an in vitro study. Br J Orthod 18:29-35.

Sunna S, and Rock WP. 1999. An ex vivo investigation into the bond strength of orthodontic brackets and adhesive systems. Br J Orthod 26:47-50.

Thanos CE, Munholland T, and Caputo AA. 1979. Adhesion of mesh-base direct-bonding brackets. Am J Orthod 75:421-430.]
